# An update on mobile applications collecting data among subjects with or at risk of Alzheimer's disease

**DOI:** 10.3389/fnagi.2023.1134096

**Published:** 2023-05-30

**Authors:** Lydia Piendel, Martin Vališ, Jakub Hort

**Affiliations:** ^1^Augusta University/University of Georgia Medical Partnership, Medical College of Georgia, Athens, GA, United States; ^2^Memory Clinic, Department of Neurology, Charles University, 2nd Faculty of Medicine and Motol University Hospital, Prague, Czechia; ^3^Department of Neurology, University Hospital Hradec Králové, Faculty of Medicine, Charles University, Hradec Králové, Czechia

**Keywords:** dementia, Alzheimer's disease, mobile phone applications, technology, tracking

## Abstract

Smart mobile phone use is increasing worldwide, as is the ability of mobile devices to monitor daily routines, behaviors, and even cognitive changes. There is a growing opportunity for users to share the data collected with their medical providers which may serve as an accessible cognitive impairment screening tool. Data logged or tracked in an app and analyzed with machine learning (ML) could identify subtle cognitive changes and lead to more timely diagnoses on an individual and population level. This review comments on existing evidence of mobile device applications designed to passively and/or actively collect data on cognition relevant for early detection and diagnosis of Alzheimer's disease (AD). The PubMed database was searched to identify existing literature on apps related to dementia and cognitive health data collection. The initial search deadline was December 1, 2022. Additional literature published in 2023 was accounted for with a follow-up search prior to publication. Criteria for inclusion was limited to articles in English which referenced data collection via mobile app from adults 50+ concerned, at risk of, or diagnosed with AD dementia. We identified relevant literature (*n* = 25) which fit our criteria. Many publications were excluded because they focused on apps which fail to collect data and simply provide users with cognitive health information. We found that although data collecting cognition-related apps have existed for years, the use of these apps as screening tools remains underdeveloped; however, it may serve as proof of concept and feasibility as there is much supporting evidence on their predictive utility. Concerns about the validity of mobile apps for cognitive screening and privacy issues remain prevalent. Mobile applications and use of ML is widely considered a financially and socially viable method of compiling symptomatic data but currently this large potential dataset, screening tool, and research resource is still largely untapped.

## 1. Introduction

The global number of individuals with Alzheimer's disease (AD) including dementia, prodromal, and preclinical stages, is estimated at 32, 69, and 315 million, respectively. Together this constitutes 416 million across the AD continuum, or 22% of all individuals aged 50 and above. Considering predementia stages, the number of people with AD is much larger than conveyed in available literature (Gustavsson et al., 2022). With the rapidly aging populations in the United States and Europe, the expected number of people living with dementia at any stage by 2050 is expected to be at 152.8 million (GBD 2019 Dementia Forecasting Collaborators, 2019).

It is well-known that the onset of AD is preceded by 5–10 years of subtle cognitive decline which may be noticed by individuals or their family members but is often compensated for until a diagnosis of mild cognitive impairment (MCI) is made and the individual has lost independence. Patients with MCI are often the target populations for candidate drugs and other AD clinical trials, although there has been little success in reversing what cognitive impairment already exists. Thus, prescreening for MCI progression is important for finding subjects for clinical drug trials, early identification of subjects for currently available treatment, and proper allocation of resources to prevent congestion of the health care system.

Mobile devices have been used in the healthcare context for years (Weir et al., 2014). The use of mobile devices in healthcare and research data collection began in the 1990's with the use of personal digital assistants (PDAs) and smartphones by healthcare workers (Mosa et al., 2012). It was reported that 30% of US physicians used smartphones in 2001, and 64% of physicians in 2009 (Mosa et al., 2012). With the onset of the COVID-19 pandemic in 2020 it became clear that cell phones can offer an extraordinary potential for more regular patient-doctor interfacing. There is growing use of mobile smart phones by older populations, and an estimated 37% of adults communicated with their physicians via telehealth in 2021 (Lucas and Villarroel, 2022).

Projects around the world are emerging to take advantage of the large amounts of data collected daily by the technology we use and wear, including mobile phones, digital watches, heart rate monitors, and sleep bands. The passively collected and actively inputted data has great potential to be analyzed using machine learning (ML) and artificial intelligence (AI) to detect subtle changes in a person's symptoms, including vocal inflection and eye gaze, which can be linked to known AD biomarkers (Harms et al., 2022). With the advent of blockchain technology, the encryption and data structuring within this format is being tested to offer more privacy and to store massive amounts of information for analysis (Hort et al., 2021).

As of 2021 more than 325,000 smartphone applications related to health were publicly available, which included those for tracking health related data, i.e., counting number of steps and monitoring heart rate (Minen et al., 2021). Despite the high number of apps that exist and continue to be released related to cognition, mindfulness, and health tracking, very few are specific to AD or even dementia in general (Minen et al., 2021). Due to the slow progressive nature of AD, and the subtleties associated with its disease progression, there is a unique potential for longitudinal screening by mobile phones apps.

With this paper we aim to comment on existing scientific literature in the PubMed database that discusses mobile phone applications which are engaged in data collection specific to detecting or tracking symptoms related to AD. We also include a brief description of some existing projects and those in development for the use of technology for big data collection related to dementia screening at large.

## 2. Methodology

A PubMed search was framed using the PICO format (Richardson et al., 1995) and conducted using the following term, utilizing the Medical Subject Heading (MeSH) terms: *[“Dementia”[MeSH Terms] AND “Mobile Applications”[MeSH Terms]] AND [english[Filter]]*. The MeSH term “dementia” was used due to its encompassing nature and the possibility of a paper referring broadly to cognitive symptoms. Our ultimate focus is on tracking symptoms related to AD specifically. Those papers on apps tracking Huntington's disease and Parkinson's disease symptoms were excluded. Results focused on papers discussing adult users over 50; this age cutoff was decided due to dementia-focused research often being inclusive of older adults 50+ and our goal to understand apps designed for the older adult population, who may not be technologically savvy and therefore require apps designed for ease-of-use. This framework and the inclusion/exclusion criteria are shown in Table 1.

**Table 1:** The inclusion/exclusion criteria in a loosely generated PICO format for the PubMed search of literature related to dementia data tracking using mobile phone apps.

**Inclusion/exclusion criteria**	
P: Population	Study or article should mention populations 50+, older adults concerned of, at risk of, or diagnosed with MCI, AD, or broadly referred to with “dementia”
I: Intervention	Study or article must mention use of a mobile phone application or mobile device that is specifically designed for use in the population as defined in “P”
C: Comparison	None
O: Outcome	Study or article must mention collection/tracking/input of user generated data relating to cognition via the mobile phone application either passively or actively
Filters:	English; all study designs/reviews/articles included

**Figure 1:**
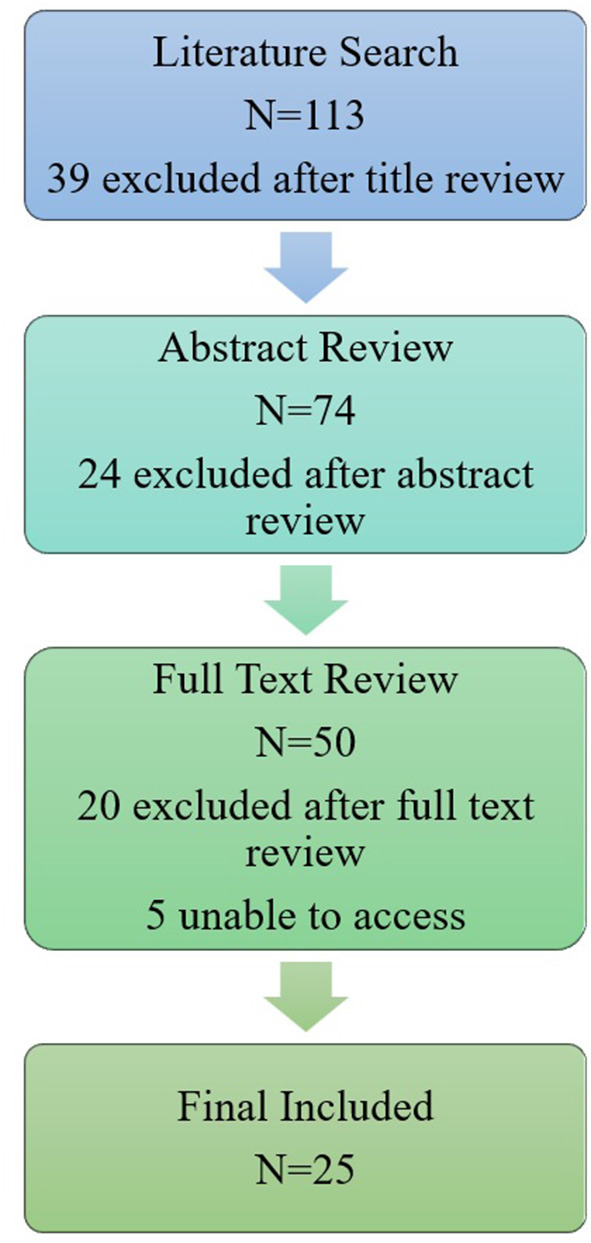
A concept map of the screening methods for including literature related to the collection of data. Our initial literature search yielded 113 results, 39 of which were excluded due to title irrelevance. Title relevance was determined due to the presence of explicit reference to one of three main categories for exclusion: animal modeling, apps exclusively developed as dementia support for caregivers, or papers on other diseases (i.e., HIV). If a title did not explicitly state a focus outside of our scope, the paper was included for abstract review. Out of the 74 abstracts reviewed, 24 results were deemed irrelevant and 50 total articles were reviewed in full if available. An abstract was excluded if the following criteria were not met: papers must have mentioned cognition, cognitive health, or dementia, and the use mobile phone application(s) by older adults at risk of or diagnosed with MCI or AD. Papers were excluded at full text review in the absence of discussion of a mobile phone application that collected data from the user, or if the paper focused on a specific dementia other than Alzheimer's disease. The final analysis of included publications is discussed in Table 2.

This search yielded a total of 113 results. Initial literature searches were concluded on December 1, 2022. Prior to publication an additional two papers were identified in the search which had been published prior to April 1, 2023. A total of 50 papers were included in the full-text review. A concept map of the screening methods is shown in Figure 1. Additional information was found using a wider internet search and references of included publications.

Results from the literature searches were screened for title relevance and then passed through an abstract-review stage in which included papers must have mentioned cognition, cognitive health, or dementia, and the use mobile phone application(s) by older adults at risk of or diagnosed with MCI or AD. Papers at full-text review were excluded if there was no mention of any capability of the mobile phone application to collect data from the user, either passively or actively. Papers were excluded if “dementia” specifically referred to a condition other than Alzheimer's disease. This review was exempt from IRB approval as the data collected were publicly accessible via PubMed and no human subjects were included.

## 3. Results

We found 25 papers which fit our inclusion criteria.

A significant number of articles found through the literature search (more than 70% of the results) were excluded due to their mention of apps which do not collect data but rather only function to share information with the user, usually a caregiver. Based on our search and exclusion of so many papers, it can be reasonably estimated that most of the existing app market (directed at people at risk of or living with dementia) is comprised of apps which do not collect data or are focused on screening. Instead, they only share information with people with dementia, caregivers, or otherwise act as assistive technology. There were several papers which wrote about the use of apps for monitoring symptoms in people with Parkinson's disease and Huntington's disease specifically, due to their clear detectable symptoms such as movement changes. These papers were excluded from our final review.

Table 2 summarizes the literature types included after full-text review which made mention of data collection from adults aged 50+ and in the context of AD or cognitive screening for AD. We broadly categorized the literature into papers which discussed (1) clinical trials or design schemas for single apps (*n* = 11), (2) literature reviews or multi-app analyses (*n* = 12), and (3) commentary/articles (*n* = 2).

**Table 2:** A summary of the literature included from the PubMed search in the full text review (*n* = 25) (Mandal et al., 2015; Zorluoglu et al., 2015; Zylstra et al., 2018; Leorin et al., 2019; Sabbagh et al., 2020; Yousaf et al., 2020; Boyd et al., 2021; Li et al., 2021; Paddick et al., 2021; Hackett et al., 2022).

**Type of literature**	**#**	**Apps featured**	**Main themes**	**Date**	**App target audience**	**Summary**
Clinical Trial or Single App Design Scheme	11	• AltoidaML • MyMindCheck • InspireD • NPI • SmartPrompt • NMI • Alzheed • MCS	• Therapy/Treatment (1) • App Design/Quality (3) • Monitoring Symptoms (3) • Screening (4)	• 2022 (2) • 2021 (5) • 2020 (1) • 2019 (2) • 2015 (1)	• PwD (7) • Caregivers (4) • Healthcare professionals (1) • MCI (3) • Older adults/controls (6)	Tracking use of apps in PwD; Co-design is important for use/adherence; Potential measurement of longitudinal symptoms; Tracking eye-gaze (2); CV risk factor improvement in individuals at risk; Apps are pragmatic; audio recordings, gait, sit-to-stand tests; monitoring BPSD progression; tapping speeds/gait/voice; Screening apps in rural areas; Reminder apps usability; Machine learning for long-term prediction models of MCI progression; Privacy risks mentioned consistently
Literature Review or Multi-App Analysis	12	• Dementia Talk • Alzheimer's Manager • CogSelfTest • BrainCheck Memory • BrainTest • MemTrax • Minstrong Health • NeuraMetrix • Altoida • DementiaTest • ConsultGeri • MCS • eSLUMS • Mental Health Test • iVitality • Color-Shape Test • DelApp • MOBI-COG • Dementia Screener • Edinburgh Dementia App	• App Design/Quality (2) • Screening (4) • Monitoring Symptoms (5) • Privacy (2)	• 2022 (2) • 2021 (1) • 2020 (2) • 2019 (2) • 2018 (2) • 2017 • 2015 • 2014	• Older Adults (3) • Caregivers (3) • PwD (7)	Most cognitive games on commercially available apps are not associated with clinical research; Many apps are available for caregivers but most do not have tracking abilities; Apps designed for PwD are often difficult for users due to cognitive impairments; Privacy and data collection mentioned consistently; Great potential for mHealth but current shortage of apps for PwD; Screening potential for cognitive impairment is great with mobile apps but validation is difficult, technological fluency is a concern; Passive monitoring is a potential screening tool via wearable technologies, speech detection
Article	2	n/a	• Monitoring Symptoms (1) • Potential App Design (1)	• 2020 • 2019	PwD (2)	AI shows accuracy in detecting speech changes between AD and control groups; Privacy is a concern; Co-design of apps with users and professionals is important

Search inclusion criteria are listed in Table 1. Some articles had more than one main theme and multiple target audiences. People living with Dementia (PwD). Cardiovascular risk (CV).

The papers in the first category of single-app discussions primarily focused on the quality of the app being designed and the use of an app for screening and monitoring symptoms in clinical trial situations. Only one app was more focused on prevention and treatment. The paper writes about the design of an app to improve cardiovascular risk factors to prevent dementia among people with multiple dementia risk factors (PRODEMOS trial, Eggink et al., 2021). Two included papers write about the use of eye-tracking technologies to determine attention span and potentially correlate to cognitive decline. Two of the studies in this first category have explicit co-design in their app development, both with experts and prospective users to promote use and adherence, especially for tracking symptoms. Three additional studies in this category write about the necessity to include usability feedback in future designs and adjustments to the apps. A main concern brought up in all five of these papers was that many apps are not designed with functional ability or usability in mind for people who are already experiencing some impairment in function. One trial discusses the use of an app to measure behavioral symptoms in moderate to severe dementia cases, and the researchers subsequently compare the patients' NPI scores with the app's tracking accuracy (Rangseekajee et al., 2021). Rai et al. write about an app, Altoida ADPS, which assesses augmented reality tasks of daily living in users with MCI and can predict conversion to AD when verified against clinical data (Rai et al., 2020). Six of the 11 papers discuss apps which have been validated in pilot trials and clinical trials when compared to clinical data but are not used in public or widespread practice. The other five papers discuss apps which are in the development phases and have yet to be brought to trials. Out of these 11 papers, only three explicitly describe how the data collection is handled concerning the maintenance of patient/user privacy. Finally, from this first category of single-app papers, most (*n* = 8) acknowledge data input from the caregiver is either solely or jointly necessary to facilitate ease of use of the app by the patient or individual with cognitive impairment.

From the second category of papers, primarily literature reviews and multi-app analyses, a main theme was the review of apps which monitor and screen symptoms longitudinally among people with MCI. This included a paper about publicly available game-based apps for smartphones which generally test all categories in the MMSE (Sabermahani et al., 2022) as well as a paper reviewing available apps with tracking features (Werner et al., 2022). It was widely written (*n* = 6) that apps which are available for public download are not all validated against cognitive testing used in clinical settings. Additionally, many of these reviews reference positive aspects of widely available screening via technology: improved patient care, earlier and faster screening, and empowerment of patients in their own care. One paper brings up an interesting and important point about bias of screening apps toward cognitively healthy individuals who are more likely to use apps in the first place (Thabtah et al., 2019). Of the 12 papers in this category, *n* = 8 discussed privacy to a large degree as an issue in app development and use, and *n* = 6 discussed caregiver input of data into apps.

The two articles included write about how AI can accurately detect speech changes in people with AD vs. control users, and that it is important to co-design apps with professionals and users in order to effectively produce an app.

In addition to the PubMed search, we found ongoing projects through internet searches of the references and app development teams from our initial search, discussing apps associated with tracking and data collection in people at risk of dementia. In Table 3, we include an inexhaustive list of some of these ongoing projects.

**Table 3:** An inexhaustive list of new and ongoing mobile phone app projects related to collecting data for cognition and dementia screening (Polzer and Gewald, 2017; Lancaster et al., 2020; Tak, 2021).

**Project/app name**	**Setting**	**Developers (academic/private sector)**	**Target audience**	**Project/app goals**	**Availability (date)**
AlzheimerChain	Mobile app. Czech Republic; Germany, Switzerland, Austria, UK, Nordic countries	AlzheimerChain Foundation	Adults; not yet available in English	Screening and verbal tests; AI evaluation of MRI; financial reward for participants	Est. 2020; App to be released 2023
Dakim	Paid application for PCs, Macs, and iPads; USA	Dakim, Inc.	Adults 50+ and 70+	Web-based brain training program	First commercially available in 2006; website last updated 2018
Sea Hero Quest	Mobile app. UK; available worldwide; data considered from 63 countries	Alzheimers Research UK, GLITCHERS by Deutsche Telekon, UCL, University of East Anglia	All ages	Spatial navigation; real-time data collection for researchers	2016; no longer available for public use; currently code protected for private research use
DelApp	Mobile bedside app for use in hospitals; UK	Edinburgh Delirium Research Group, University of Edinburgh, UK	Patients 65+ with delirium or otherwise unable to be assessed by formal cognitive testing	Accurate and fast determination of delirium severity vs. other cognitive impairment	Official study published 2018 (Rutter et al., 2018)
DailyCog	Mobile app. For use in the Android system. Israel.	Israeli Ministry of Science and Technology; University of Haifa, Israel	Patients with MCI in Parkinson's disease	Detecting and evaluating MCI in early Parkinson's disease via short tasks in the app and self-reporting	Presented in 2019, additional study completed in 2021
Altoida	Mobile app. USA; available worldwide	Altoida (private company) supported by both commercial and academic entities; i.e., Lilly, NYU Langone	Older adults	Using augmented reality to measure 13 neurocognitive domains for early dementia detection	In development since 2001; major Nature publication in 2020
EDoN	Digital devices (i.e., smartwatches, apps, headbands); UK	Alzheimers UK	Older adults	Utilizing mobile apps and health monitoring devices for large-scale identification of people at risk of dementia	In development since 2019 (Frey et al., 2019)
Mezurio	Mobile app. UK; available worldwide	Oxford University Big Data Institute; funded by Roche, Eli-Lilly, Robertson Foundation; UK Alzheimer's Society	18+ without dementia; Adults 40–59 years with no cognitive impairment	Episodic memory, executive function, and language tasks	2019; Code protected for private research use

## 4. Discussion

Several themes emerged in the review of literature on mobile applications for Alzheimer's disease screening and tracking. We explore these in the following Discussion section.

### 4.1. Apps are generally designed for caregivers

One common theme regarding mobile applications for use in AD screening is the inaccessibility of use by people with low technological literacy and/or cognitive impairment. Therefore, most apps developed are designed for caregivers, rather than those with the disease. In our search we included papers which made mention of use by the person with or at risk of impairment when possible. Many apps discussed in papers included in our search did allow caregivers to input data due to severe cognitive decline; these papers were included in our search as they focused on the use of an app for tracking purposes. Ultimately more than 20% of papers from our literature search were excluded from our review as they explicitly focused on supportive mobile applications available to caregivers of people with dementia; for example, the app giving a caregiver information about AD, rather than apps made for collecting data from people with or at risk cognitive impairment. We found this to be consistent with previously written reviews on dementia mobile applications. For example, there is a team in Australia that conducted a systematic review of mobile applications related to dementia or Alzheimer's information, screening apps with the mobile application review system (MARS). Reviewing 75 apps, the authors found that most were targeted at caregivers and that the content primarily related to providing information and resources rather than any data collection (Chelberg et al., 2022). Among the included papers in our search, a strong theme was the App Design/Quality and co-design of apps by users, for example caregivers and people living with dementia (PwD), as well as app designers and healthcare professionals (Fox et al., 2022).

### 4.2. Mobile screening apps validated with cognitive tests are primarily used in clinical trials

Our final search included 12 review papers that together searched hundreds of apps related to dementia, and explicitly mentioned more than 20 unique screening apps as shown in Table 2. The existence of such reviews is evidence of the ever-growing relevance of mobile technologies in AD dementia screening and the value in investment of these technologies. However, one major concern is the validity of screening tools used in uncontrolled environments, should this technology be introduced for public use. In addition to the review papers, we considered 11 articles in our final search which presented designs and pilot trials for specific apps based in validated environments and compared to neuropsychiatric tests such as the Montreal Cognitive Assessment (MoCA) or Neuropsychiatric Inventory (NPI). These groupings show that apps available for public use are most often separate from those validated against cognitive tests, which must be subject to ethical approval, HIPAA concerns, and controlled environments.

A few reviews provided an overview of the mobile applications based in clinically verified cognitive testing; one of which was conducted by Thabtah et al. (2020). This systematic review identified mobile phone applications used as dementia screening tools by individuals, caregivers, or clinicians, and the authors included only apps which aligned themselves with specific diagnostic criteria in neurocognitive domains in the Diagnostic and Statistical Manual of Mental Disorders, Fifth Edition (DSM-5). Through their search via Google, Android, and iTunes they identified 275 applications related to dementia but excluded a majority because they did not include screening tools. Their final analysis resulted in 20 applications being reviewed based on their screening assessments. This review and many others did not comment on how the data from these apps were being used, how data were stored, and who had access to it. This begs the question of what the data could and should be used to accomplish. Out of the 20 applications Thabtah et al. (2020) summarized, only one app (ACE) was targeted for health care professionals; all the other apps were generally targeted at non-medical professional adult users in the general public. Still, many of the apps they reviewed did have the option to produce a formal medical report to be given to a clinical or shared via electronic mail. Finally, they discuss the potential of machine learning as a process to learn from the data collected and potentially identify and improve accuracy of screening, but write that only a few existing apps actually use artificial intelligence (AI) in identifying dementia risk (i.e., Cognity identifies risk based on a clock drawing and its comparison to a larger dataset).

An example of a clinical trial we included in our final search was published by Rangseekajee et al. (2021) in Thailand. Their team conducted a study to validate an app they developed to measure neuropsychiatric symptoms. The target audience for application use is caregivers of those with dementia, who regularly see PwD and can input behavioral and cognitive observations on a weekly basis. This team developed the app based on a digital modification of the NPI and found that the mobile version was 93% valid against paper and pencil NPI scores. The team also found that 80% of caregivers reported that the app was “very likely to be helpful in caregiving” supporting the notion that mobile applications are accessible, consistent, and valid.

Several conclusions can be drawn about the public vs. professional use of applications collecting screening data. First, the use of apps in controlled clinical trial settings is well-supported and indicates the movement of the AD field toward continued use of mobile technologies and AI as screening measures for early detection as a supplement to clinical testing or other biomarker testing. Second, as mobile phone applications continue to be developed for public use, more work should be done to create apps which are functionally accessible to potential users given their age and potential cognitive status, and to create apps which are validated in external home environments.

### 4.3. Many systematic reviews of apps are outdated and not focused on data collection

Although the papers we reviewed identified single phone applications or in the context of a mobile application store search, none of the reviews aimed to find the outcome of the data collected by the apps. Additionally, only 2/12 reviews we included in our final set have been published in the past year. One of those, as part of a PhD dissertation, focused on cognitive smartphone games relating to domains of the Mini Mental State Examination (MMSE; Sabermahani et al., 2022). The other reviewed 17 apps designed for caregivers and found only 2/17 included tracking features: Dementia Talk and Alzheimer's Manager (Werner et al., 2022). Mobile phone usage is a dynamic field and new apps are developed regularly. Continuing to evaluate what applications are available for PwD and what data may be tracked via these apps is crucial as technology continues to improve.

One paper excluded from our final set due to no mention of data collection was a systematic review by Brown and O'Connor (2020) on mobile apps for people with dementia, focusing on a review of research studies and asking two main questions about a person with dementia's experience using a health app, and then which factors influence the implementation of an app for a person with dementia. They included nine studies and found that mobile technology used for people with dementia primarily serves to stimulate cognition and communication. This paper serves as an example of an app review which does not mention the use of mobile health applications for data collection or research purposes.

Another review paper we did not include in our final set, published in 2020, looked at mobile phone applications related to dementia that either had a primary function of assisting patients with certain needs or that had a primary function of educating patients with needs of dementia care (Guo et al., 2020). They aimed to analyze the benefits of the applications, the content, and the quality of features available. From an initial search result of 245 apps between the app stores, they narrowed this down to 14 apps which met their criteria. They found that the available apps in 2019 were not meeting the complex needs of people with dementia and their communication impairments. This review did not focus on the outcomes of the applications from a data collection capability standpoint, but rather the educational potential. They found that most of the apps focused on general education tips, alerts, and social networking. Although not focused on neuropsychiatric symptoms or cognitive and functional aspects of dementia, the authors suggest that the use of mobile technology is so complex and intertwined with our daily lives that it will likely have “extensive influence on health care” including in the communication sphere of sharing information between patients and their health care professionals.

### 4.4. Machine learning and AI are used for tracking other diseases and making predictions

Multiple articles we found featured clinical trials of apps tracking symptoms of Huntington's disease, Parkinson's disease, and delirium (Tieges et al., 2015; Chinner et al., 2018; Cohen et al., 2018; Waddell et al., 2021). Additionally, a large majority of health-related apps available in the marketplace are related to mental health such as depression and anxiety (Minen). These are just a few examples of cognitive related disorders which manifest in symptoms that may be easily trackable via mobile application and detectable using AI. These symptoms can include gait, sit-to-stand changes, eye gaze, attention, tremor, and vocal intonation.

An article published in Nature in 2020 highlights this concept as the author describes how developers of vocal analysis using machine learning and AI have long been considering this technology for screening of diseases such as COVID-19, flare-ups of COPD, and Parkinson's disease. The “vocal biomarkers” like speech distortions, shortness of breath, and weakness in speech could be detected earlier via AI; each are distinctive features of these diseases. Although vocal changes are not historically known to be prominent symptoms of AD, one group of researchers at the University of Toronto was able to use machine learning to correctly identify people with AD with 92% accuracy by analyzing other features of speech, such as the use of fragmented sentences (Anthes, 2020).

Many clinical trial publications we found (see Table 2) focused on the development of single apps which track changes in PwD and those changes are compared with clinical disease progression. One such app, called Alzheed, was developed for use by healthcare professionals at a day center for AD. The authors write that machine learning models are not yet available, but planned to be built for determining treatment decisions in the PwD (Chávez et al., 2019). Another author (Serra-Añó et al., 2019) writes that the sensors in Android devices are already able to use motion data to differentiate functional abilities in people with AD vs. a control group.

An additional clinical trial publication (Rai et al., 2020) discusses a prognostic model using an app (NMI) with machine learning to predict long term symptomatic progression of MCI and conversion to AD, primarily using spatial navigation related augmented reality tasks. The spatial navigation features they mention include screen touch frequency, reaction time, and eye tracking and for each app user, a report is generated and sent to the study physician in a HIPAA compliant manner. When compared to clinical progression, the machine learning models they used proved to be quite successful (Rai et al., 2020).

One conclusion we can draw from our search is that mobile technology and phone applications have been successfully developed for tracking cognition-related disease, indicating space and potential for these technologies to detect features of dementia earlier and more accurately in the future, whether that includes the use of passive phone apps tracking vocal changes, movements, eye gaze, or perhaps other subtleties that only a tracking device could perceive.

### 4.5. Privacy is a big issue

Some authors conducting clinical trials or reviews about mobile applications are aware of the privacy issues related to this technology; for example, three of the 11 papers we categorized into single-app discussions explicitly wrote about integrating patient privacy into their designs. As the author of the Nature article states, without proper regulation, these AI technologies could be used for potential discrimination by insurers and employers, and individuals may be falsely diagnosed, or their information may be sold or abused (Anthes, 2020).

Two of the reviews we found focused primarily about privacy concerns and mobile health apps. Rosenfeld et al. (2017) reviewed 72 apps for PwD and found that most do not offer clear or any privacy policies for users. Four years later, Minen et al. (2021) reviewed 83 apps created for five different major neuropsychiatric conditions (including AD/dementia) and they found once again that there are few privacy policies available and almost no regulation via HIPAA about the data obtained in these apps, unless the app is privately used and password-protected for participants in clinical trials.

A review from 2019 systematically searched for free mobile phone applications which collected potentially identifiable screening data related to cognition and brain stimulation. The authors found that a majority of apps reviewed (78%) do not have an available privacy policy, or terms and conditions listed. The authors write that applications designed solely for brain training often request personal information, but that there is no need for identifiable information input unless the applications then allow data to be shared with health professionals, which they largely do not. Apart from this, there is no other mention about the large-scale collection of this data which could be used for larger research purposes (Muchagata, 2019).

## 5. Ongoing projects and conclusion

As we have explored, AD is an ideal disease target for the use of mobile phone applications and passively collected digital biomarkers because the onset of the disease symptoms is thought to be 10–15 years after the initial disease pathology begins undetected in the brain. The use of AI and machine learning could detect slight changes in a person's use of technology, leading to earlier detection. Today most adults actively carry and use a mobile device; using an app to collect passive and active data from these users over an extended time has potential for researchers, clinicians, and the public.

Through our search we identified limited literature (*n* = 11) that focuses on single-app development and case studies or small group testing of dementia apps which also collect data. All but one of these articles were published between 2019 and 2022, suggesting this is a very new and upcoming field. In addition to the papers we found via literature search, we have also identified other ongoing dementia app projects (Table 3).

A clinical group in Israel developed DailyCog, an app to collect information on MCI-PD patients (Rosenblum et al., 2021). Their app collects both user inputted data (i.e., answers to questions) as well as background “hidden markers” including the amount of time spent answering each question or time spent going between screens. The authors mention that this data is “logged for future analysis” but do not mention who would have access to this data and if it could be used for identification of potential at-risk dementia or Parkinson's disease subjects for large scale prevention trials.

One well-known project taking advantage of AI in neurology is called Altoida (Harms et al., 2022), which aims to identify digital biomarkers of MCI and AD, focusing on sex-based differences in these biomarkers. As reported by Harms et al. (2022) and Livingston et al. (2020), ~ 40% of dementias are estimated to be a result of modifiable risk factors. Therefore, the early detection of risk for dementia could lead to initiation of protective treatments, lifestyle changes, and other modifications to prevent or lessen the risk. The Altoida project is an example of the potential in big data collection for AD screening and research purposes. They have already published validated studies with prototype apps and secured more than $20 million in funding to develop and implement a smartphone compliant mobile app that will use virtual reality to identify behavioral and functional changes in users through their interactions in a simulated daily environment. Their platform will produce test results that could identify people at greater risk; however, it is not yet clear how the data will be used and to whom it will be accessible.

There are many other initiatives and groundbreaking scientists and collaborators who recognize the value and untapped potential of mobile applications tracking data for research purposes in dementia. One of those groups comes from the Alzheimer's UK initiative, called EDoN. Their project is aimed at early detection by taking advantage of the mobile technology and health tracking devices we use today, focusing broadly on data collection tracking devices (such as monitoring heart rate, sleep, cognition, and spatial navigation) by using digital devices (smart watches, apps, and headbands; Frey et al., 2019). EDoN promotes the idea that the use of mobile applications and health monitoring devices is valuable for large-scale early identification of people at risk for dementia, as it is low burden to individuals and cost-effective (i.e., rather than PET and CSF sampling). The authors are explicit in their goal for big-data collection and write:

“One of the key outputs of the project will be a wealth of curated digital and clinical data. Openly available software and metadata, combined with a transparent data access policy, will allow researchers to investigate the earlier stages of dementia-causing diseases, enabling the examination of preventative measures, treatments and causes of disease” (Frey et al., 2019).

Our review has limits. First by nature of this quickly evolving field, there are ongoing projects under development with strong back-up which are likely to launch mobile apps by the time this review has been published. We also have covered papers written in English only which may have excluded papers or projects being developed in other languages. The search term utilized additionally limited our search to modern papers which included the term “application” and older game-related papers may not have been included, as papers have previously utilized the term “smartphone” and “games” rather than “applications.”

Our systematic literature search and associated internet searches of mobile phone applications related to dementia led us to find a limited number of existing apps which are used to track regular progression of memory, cognition, neuropsychological profiles, and behaviors. Today it is common for adults around the world to use and engage with their smartphones daily. By collecting data from a variety of users in an application format, there is potential to gather a diverse data set, digital biomarkers, and other possible screening tools to identify people at possible risk of developing dementia. This review was conducted to identify a gap in the field about which apps could be used to track data. Individuals who are at risk or concerned about dementia development may prefer the use of a mobile phone application to in-person screenings for its ease, affordability, and accessibility. This could allow for much more widespread and equitable screening for cognitive deficit. Additionally, there may be discrepancies between what is seen in clinic vs. what is happening in an individual's progression at home. At this time, most publicly available related applications do not collect regular, reliable data that can be used in research and in clinical settings, and big data generating apps are still in development. Importantly, any future in this field must include safeguards for reliability and validity of the information collected, plus data privacy measures. Ultimately, we found that the use of mobile phone apps for caregivers and people at risk or with suspected MCI and dementia is an evolving field with great potential for use as a telemedicinal data collection tool.

## Data availability statement

The original contributions presented in the study are included in the article/supplementary material, further inquiries can be directed to the corresponding author.

## Author contributions

LP conceptualized, researched, and wrote the paper. JH provided input on the conceptual aspects. JH and MV provided edits and mentorship. All authors contributed to the article and approved the submitted version.
